# Sickle Cells Abolish Melanoma Tumorigenesis in Hemoglobin SS Knockin Mice and Augment the Tumoricidal Effect of Oncolytic Virus *In Vivo*

**DOI:** 10.3389/fonc.2016.00166

**Published:** 2016-07-08

**Authors:** Chiang Wang Sun, Candice Willmon, Li-Chen Wu, Peter Knopick, Jutta Thoerner, Richard Vile, Tim M. Townes, David S. Terman

**Affiliations:** ^1^Department of Biochemistry and Molecular Genetics, University of Alabama Medical School at Birmingham, Birmingham, AL, USA; ^2^Department of Molecular Medicine, Mayo Clinic Foundation, Rochester, MN, USA; ^3^Department of Immunology, University of North Dakota Medical School, Grand Forks, ND, USA; ^4^Hisotpathology Section, Hospital of the Monterey Peninsula, Monterey, CA, USA

**Keywords:** sickle cells, melanoma, neoangiogenesis, oncolytic viruses, reactive oxygen species

## Abstract

Insights from the study of cancer resistance in animals have led to the discovery of novel anticancer pathways and opened new venues for cancer prevention and treatment. Sickle cells (SSRBCs) from subjects with homozygous sickle cell anemia (SCA) have been shown to target hypoxic tumor niches, induce diffuse vaso-occlusion, and potentiate a tumoricidal response in a heme- and oxidant-dependent manner. These findings spawned the hypothesis that SSRBCs and the vasculopathic microenvironment of subjects with SCA might be inimical to tumor outgrowth and thereby constitute a natural antitumor defense. We therefore implanted the B16F10 melanoma into humanized hemoglobin SS knockin mice which exhibit the hematologic and vasculopathic sequelae of human SCA. Over the 31-day observation period, hemoglobin SS mice showed no significant melanoma outgrowth. By contrast, 68–100% of melanomas implanted in background and hemoglobin AA knockin control mice reached the tumor growth end point (*p* < 0.0001). SS knockin mice also exhibited established markers of underlying vasculopathy, e.g., chronic hemolysis (anemia, reticulocytosis) and vascular inflammation (leukocytosis) that differed significantly from all control groups. Genetic differences or normal AA gene knockin do not explain the impaired tumor outgrowth in SS knockin mice. These data point instead to the chronic pro-oxidative vasculopathic network in these mice as the predominant cause. In related studies, we demonstrate the ability of the sickle cell component of this system to function as a therapeutic vehicle in potentiating the oncolytic/vasculopathic effect of RNA reovirus. Sickle cells were shown to efficiently adsorb and transfer the virus to melanoma cells where it induced apoptosis even in the presence of anti-reovirus neutralizing antibodies. *In vivo*, SSRBCs along with their viral cargo rapidly targeted the tumor and initiated a tumoricidal response exceeding that of free virus and similarly loaded normal RBCs without toxicity. Collectively, these data unveil two hitherto unrecognized findings: hemoglobin SS knockin mice appear to present a natural barrier to melanoma tumorigenesis while SSRBCs demonstrate therapeutic function as a vehicle for enhancing the oncolytic effect of free reovirus against established melanoma.

## Introduction

Insights from the study of cancer resistance in animals and humans have led to the discovery of novel anticancer pathways and opened new venues for cancer prevention and treatment (1, 2). Mammals, such as elephants, blind moles, and some feral murine strains, have evolved intricate mechanisms to eradicate cancer. These include cell-cycle accelerated DNA repair, checkpoint blockade, programed cell death, and replicative senescence controlled by a network of tumor-suppressor genes and mediators such as p53, Rb, and IFN-β (1–5). In genetically deficient humans with an increased incidence of tumors, the major defects appear in DNA repair and proapoptotic pathways (6). Recently, subjects with deficiencies in growth hormone receptor and insulin-like growth factor have shown a reduced cancer mortality highlighting a role of pro-growth pathways, oxidative stress, and age-dependent genomic instability in human oncogenesis (7) in a study of cancer incidence in more than 16,000 patients with sickle cells anemia (SCA), melanoma was not listed (8). To date, the notion that SCA or an animal model thereof might constitute a natural barrier to melanoma tumorigenesis has not been explored or demonstrated.

Sickle cell anemia is a monogenic hemoglobinopathy wherein glutamic acid, the sixth amino acid in the β-globin chain, is displaced by valine (9, 10). This results in hemoglobin polymerization and sickling morphology during hemoglobin desaturation. Clinically, the disease is characterized by chronic hemolysis, intermittent vaso-occlusive events, and organ injury (11, 12). Endothelial cells are chronically activated and injured after contact with sickle cells, sickle cell-derived heme, and inflammatory mediators (9–12). Contributing to the microvascular injury and dysfunction are abundant reactive oxygen species (ROS) generated in the course of relentless ischemia–reperfusion, chronic hemolysis, and vascular inflammation (13–21).

The Townes model of SCA in knockin mice faithfully replicates the clinical sequalae of human homozygous SCA together with its pro-oxidative and thrombo-inflammatory microvascular features (22–25). Such mice exhibit a γ–β^S^ globin configuration wherein mouse β-globin genes are replaced with human γ- and β^S^-globin genes and mouse α-globin genes are replaced with human α-globin genes (26, 27). They also switch human hemoglobins (HbF to HbS) at 3 weeks of age when they start to develop chronic hemolysis, severe anemia, and vascular inflammation (28).

Sickle cells (SSRBCs) from patients with SCA possess an ability to target established tumors and together with exogenous pro-oxidants produce a tumoricidal response (29). Recent studies have drawn a striking parallel between the hypoxic, pro-oxidative, and chronically upregulated microvasculature in SCA and the activated neovasculature of solid tumors (11, 22, 29–31). In both vascular networks, intravital microscopy shows that SSRBCs adhere rapidly to upregulated endothelium and recruit leukocytes and platelets forming microcellular aggregates that lead to vaso-occlusion (10, 29, 32–39). SSRBC velocity in venules of transgenic sickle mice and solid tumors is reduced resulting in SS hemoglobin polymerization and autohemolysis with consequent release of intracellular heme. The latter is a powerful cell toxin that together with ROS appear to mediate endothelial injury (14–16, 29). The consequent microvascular ischemia in SCA produces tissue injury and painful crisis while in tumors it causes tumor cell necrosis (29).

Collectively, these data spawned the hypothesis that the pro-oxidative and thrombo-inflammatory vascular microenvironment of SCA might interdict melanoma neoangiogenesis and thereby constitute a natural antitumor defense against this tumor. We therefore elected to study the outgrowth of B16F10 melanoma in transgenic mice with SCA.

In related studies, we examine the ability of human sickle cell component of the system as a therapeutic to augment oncolysis by a vasculopathic RNA virus. Previously, SSRBCs have demonstrated increased adherence to endothelium infected by RNA viruses (40). Indeed, some RNA viruses are known to target tumor neovasculature, compromise blood flow, and cause significant vascular injury (41, 42). Reovirus, a non-enveloped double stranded RNA virus, infects endothelial cells *via* its junctional adhesion molecule-A (JAM-A) and provides a conduit for the virus into the bloodstream (43, 44). In tumor cells, reovirus replicates independently of the Ras-EGFR pathway and exerts selective oncolysis *via* defective antiviral PKR (45, 46). While this agent is effective when delivered intratumorally alone or together with anti-PDI or chemotherapy, it has shown only modest ability to kill tumor when used systemically. This is largely due to binding by circulating blood cells and elimination by seroreactive neutralizing antibodies (47–51). By contrast, when the virus is adsorbed to the surface of the dendritic cells and T cells, it can be delivered to tumor niches in lymph nodes where it exhibits tumoricidal activity (52). In search of a cell carrier for systemic delivery of reovirus, we reasoned that sickle cells might be a viable candidate to protect reovirus from host defenses, target it to tumor and synergize with virus-induced tumor vascular injury. Indeed, sickle cells have been shown to carry and deliver cytotoxics into hypoxic 4T1 carcinomas and release fourfold more drug cargo into the tumor milieu than similarly loaded normal RBCs and free drug (53). We therefore loaded sickle cells with reovirus and found that the virus spontaneously translocates to melanoma cells *in vitro* and induces a tumoricidal response *in vitro* and *in vivo* exceeding that of similarly loaded normal RBCs (nRBCs) and free virus.

## Results

### B16F10 Melanoma Outgrowth Is Impaired in Sickle Cell Knockin Mice

**Figure 1:**
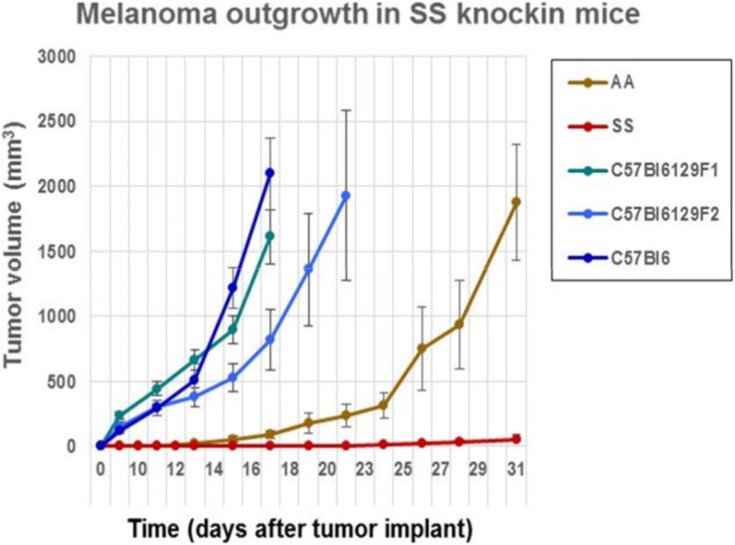
**Comparison of the outgrowth rate of B16F10 melanoma in sickle cell knockin mice with background and AA knockin mice is shown**. B16F10 melanoma outgrowth is inhibited significantly in sickle cell knockin mice compared to background and AA knockin controls (*p* < 0.0001).

We determined whether the B16F10 melanoma indigenous to C57Bl6 mice could grow after implantation into sickle cell knockin mice expressing human β^S^-globin genes and hemoglobin AA knockin mice expressing human β^A^-globin genes. These mice exhibit a background consisting of C57Bl/6 and 30% of 129 genes by QP analysis. For background controls, we deployed C57Bl/6 mice and also B6129SF1/J mice and B6129SF2/J, which express murine 129 genes along with B6 genes. Figure 1 shows that B16F10 melanoma grew robustly in C57Bl/6, B6129SF1/J mice, and B6129SF2/J mice with mean volumes of 1000 mm^3^ by days 16–18 after implantation. Tumor outgrowth to a mean of 1000 mm^3^ was also evident in the AA knockin mice by day 27. By contrast, melanoma outgrowth was impaired significantly in SS knockin mice relative to all controls (*p* < 0.0001).

**Figure 2:**
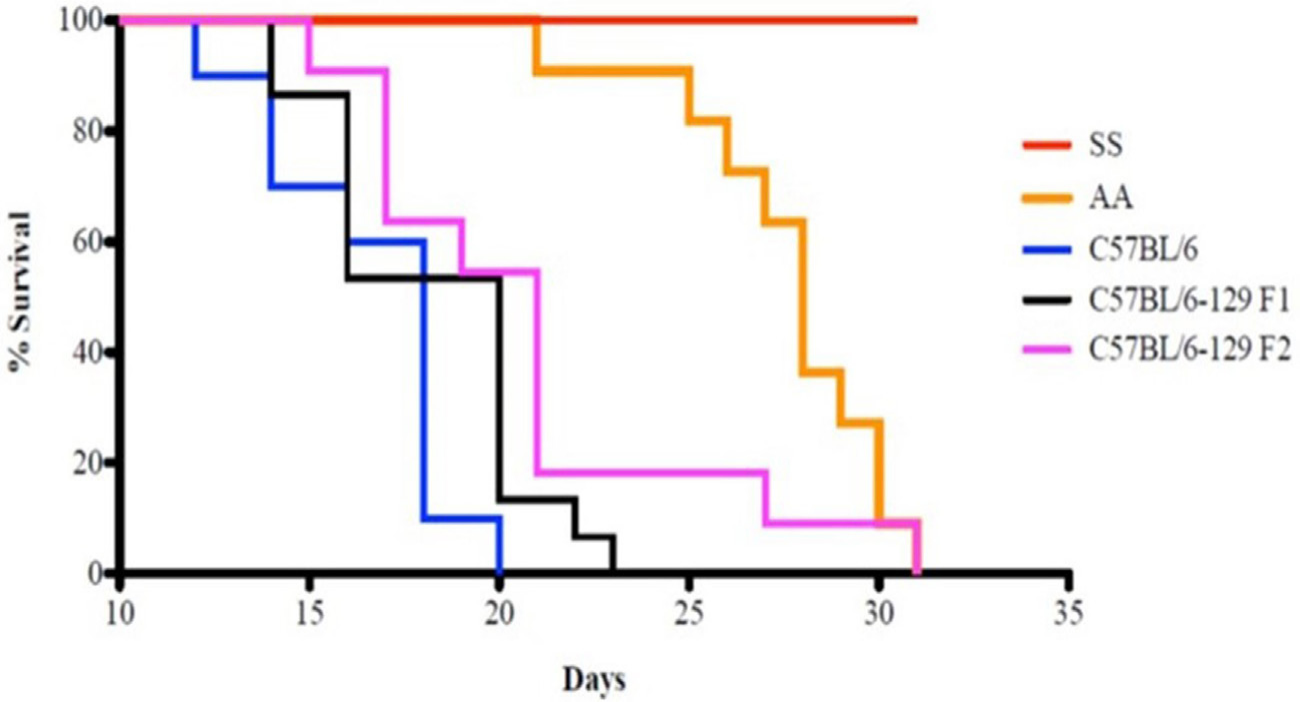
**Kaplan–Meier analysis of sickle cell mice and controls is shown**. Survival is significantly prolonged in the SS knockout mice compared to corresponding background controls and AA knockin mice. See text for details.

Kaplan–Meier analysis of survival to the tumor growth end point (tumor volume of 750 mm^3^) similarly indicates that control groups reached median end points well before day 31 after implant, whereas in sickle knockin mice, none of the implanted tumors reached this end point during this period (*p* < 0.0001 compared to all controls, Figure 2).

**Figure 3:**
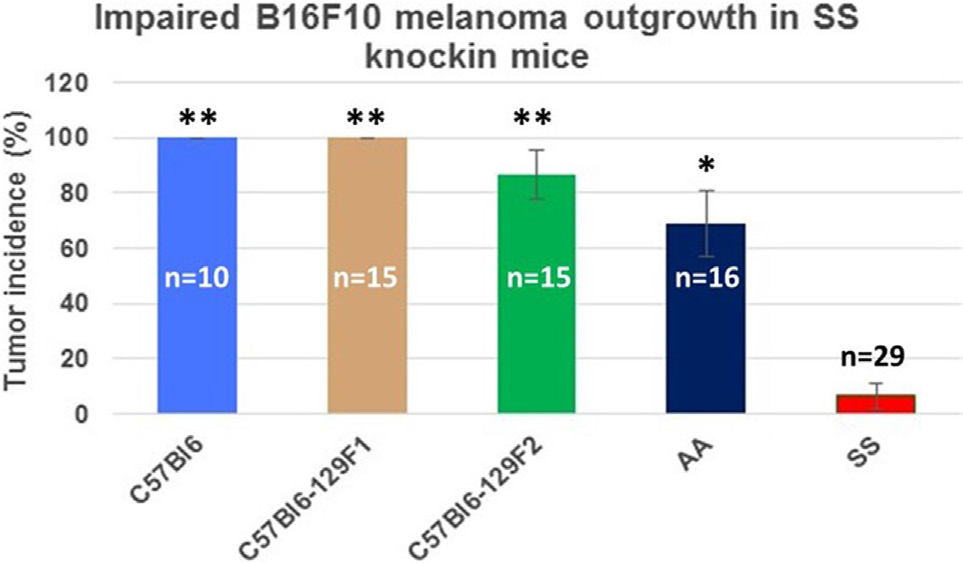
**Comparison of the percentage of B16F10 melanomas exhibiting growth to end point tumor volume within the 31-day observation period in SS knockin mice and background/allotypic hemoglobin controls is shown**. B16F10 melanoma outgrowth is significantly impaired in SS knockin mice compared with C57Bl6, C57Bl61296F1, C57Bl6129F2 (**p < 0.0001), and AA knockin (*p = 0.0004) mice.

**Figure 4:**
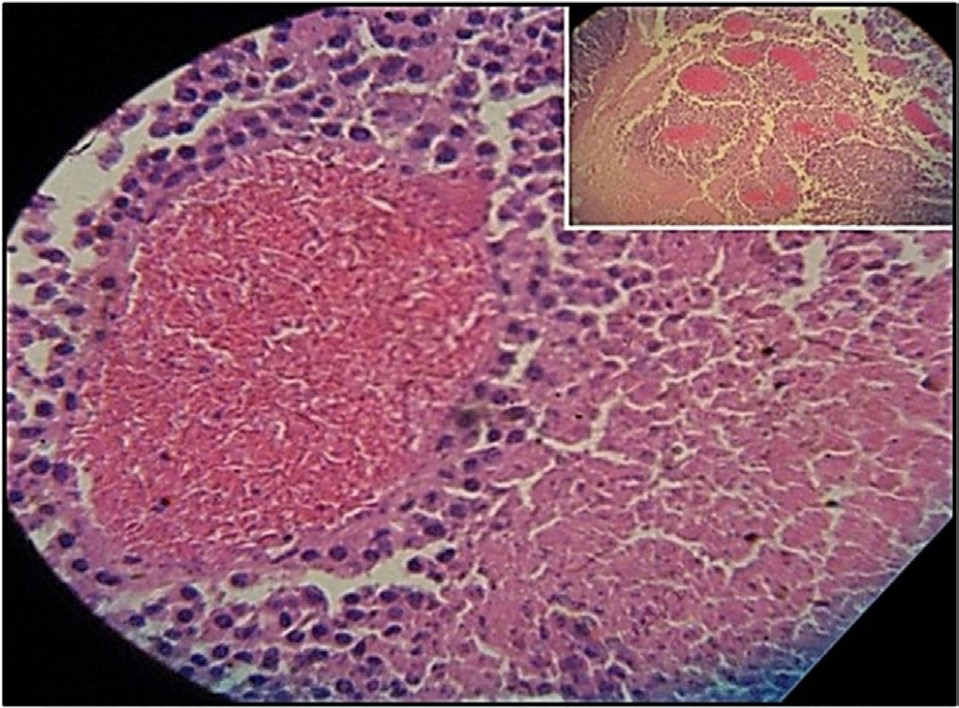
**Histopathologic section of melanoma implanted in sickle cell knockin mice that failed to show progressive growth is shown**. H&E section of melanoma implanted into SS knockin mice is shown. Insert shows cluster of eleven venules congested with sickle cells (10× magnification). Higher power shows sickle cells occluding a venule with surrounding mononuclear cell infiltrate and adjacent tumor cell necrosis (40× magnification).

Figure 3 shows the percentage of tumors that exceeded 250 mm^3^ in each group during the 31-day observation period. In sickle cell knockin mice, only 2 of 29 (6.9%) tumors grew beyond this baseline size compared to 100% of C57Bl/6 and B6129SF1/J mice (*p* = < 0.0001), 81.2% of B6129SF2/J mice (*p* < 0.0001), and 68.7% of AA knockin mice (*p* < 0.0004). Notably, the two tumors in the sickle cell knockin group that grew above baseline did not reach the 750 mm^3^ growth end point during the 31-day observation period. Histopathologic examination of the melanomas in the latter two mice showed clusters of venules congested with SSRBCs, surrounding mononuclear cell infiltrate and adjacent tumor cell necrosis (Figure 4). These results demonstrate that the incidence of B16F10 tumorigenesis is significantly impaired in SS knockin mice relative to background and AA knockin controls.

### Hematologic Evaluation of SS Knockin Mice and Controls

We examined the notion that the pro-inflammatory and pro-oxidative vascular environment of SS knockin mice could explain the impaired melanoma outgrowth in SS knockin mice. Major causative factors of such pro-oxidative microvascular milieu in SS knockin mice are unrelenting ischemia–reperfusion, chronic hemolysis, and vascular inflammation (46, 47). In these mice, anemia and reticulocytosis are established biomarkers of underlying chronic hemolysis, while leukocytosis is an indicator of sustained vascular inflammation (48, 49). We therefore compared reticulocyte, leukocyte, and hemoglobin levels of SS knockin mice with all controls. Results shown in Table 1 indicate that the degree of reticulocytosis and leukocytosis in hemoglobin SS knockin mice was significantly different from all control mice (*p* < 0.00001 and *p* < 0.0001, respectively). Likewise, SS knockin mice were significantly more anemic than all controls (*p* < 0.0001). These results indicate that unlike controls, SS knockin mice exhibit severe anemia, exaggerated reticulocyte and leukocyte counts consistent with their underlying chronic hemolysis, pro-inflammatory and pro-oxidative vasculopathy.

**Table 1:** **Comparison of biomarkers of chronic hemolysis and vasculopathic inflammation in SS Knockin mice and controls**.^a^

	SS	AA	C57BL/6
Hct	28.3 ± 4.4 (*n* = 13)	41.6 ± 2.3 (*n* = 6)	42.4 ± 5.8 (*n* = 15)
Hgb	5.9 ± 1.1 (*n* = 14)	13.0 ± 0.9 (*n* = 20	14.2 ± 3.0 (*n* = 15)
Reticulocytes	73.2 ± 6.7 (*n* = 14)	11.4 ± 6.9 (*n* = 20)	5.4 ± 1.2 (*n* = 13)
WBCs	31.7 ± 4.6 (*n* = 14)	14.6 ± 4.6 (*n* = 20)	13.8 ± 6.3 (*n* = 14)
**Comparative statistics**			
	**Hemoglobin**	**Reticulocytes**	**WBCs**
SS vs. AA	*p* < 0.0001	*p* < 0.0001	*p* < 0.0007
SS vs. C57Bl-6	*p* < 0.0001	*p* < 0.0001	*p* < 0.0002
AA vs. C57Bl-6	ns	ns	ns

*^a^Data are given as the mean and SD*.

*“n” is the number of samples tested*.

### Sickle Cells Adsorb and Transfer Oncolytic Reovirus Vector to Melanoma Cells *In Vitro* and *In Vivo*

**Figure 5:**
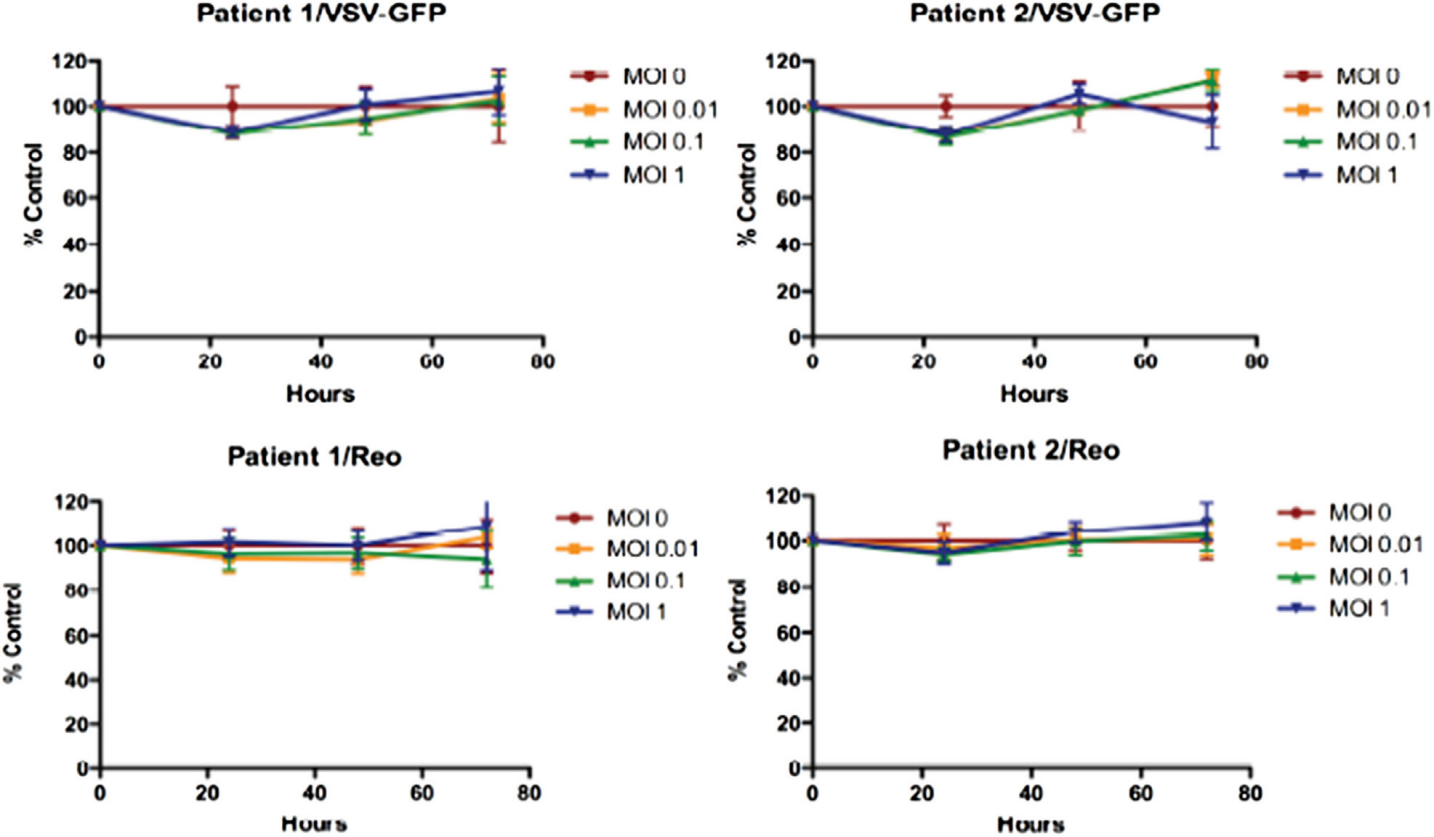
**Reovirus and vesicular stomatitis virus bind but do not replicate in SSRBCs**. Reovirus and VSV do not replicate in and are not cytotoxic to SSRBC. Human SSRBC were plated in 96-well plates and were infected with various multiplicities of infection (MOI) with reovirus and VSV. At specific time points, an MTT assay was performed according to manufacturer’s instructions. Data are plotted as a percentage of control survival with SD.

**Figure 6:**
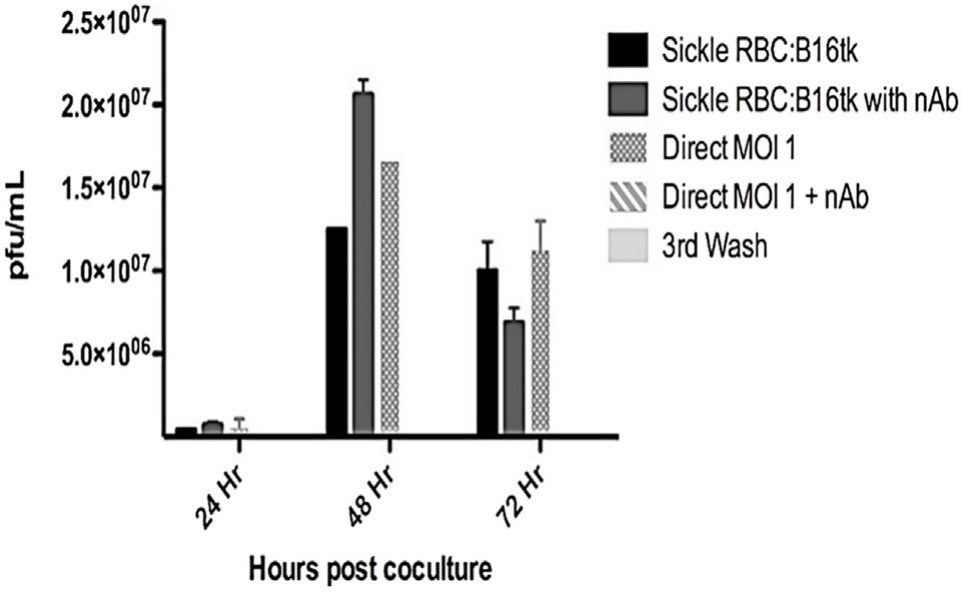
**Reovirus is transferred to melanoma cells *in vitro* in the presence of neutralizing antibody**. Shown above is B16tk melanoma cell lysis after co-incubation with SSRBCs that were loaded with reovirus (MOI = 1) for 2 h at 37°C, washed three times at a ratio of 1:1. In addition, at the time of coculture, reovirus neutralizing antibody was added to some of the wells. To demonstrate efficient removal of any unbound virus from the viral loaded SSRBC, an aliquot of the third wash was directly added to the B16tk cells. As a positive control, B16tk cells were directly infected with reovirus at an MOI of 1 with and without neutralizing antibodies. At specified time points, aliquots of the supernatant were collected and titered on L929 cells using a standard plaque forming unit assay. Data are plotted with SD.

**Figure 7:**
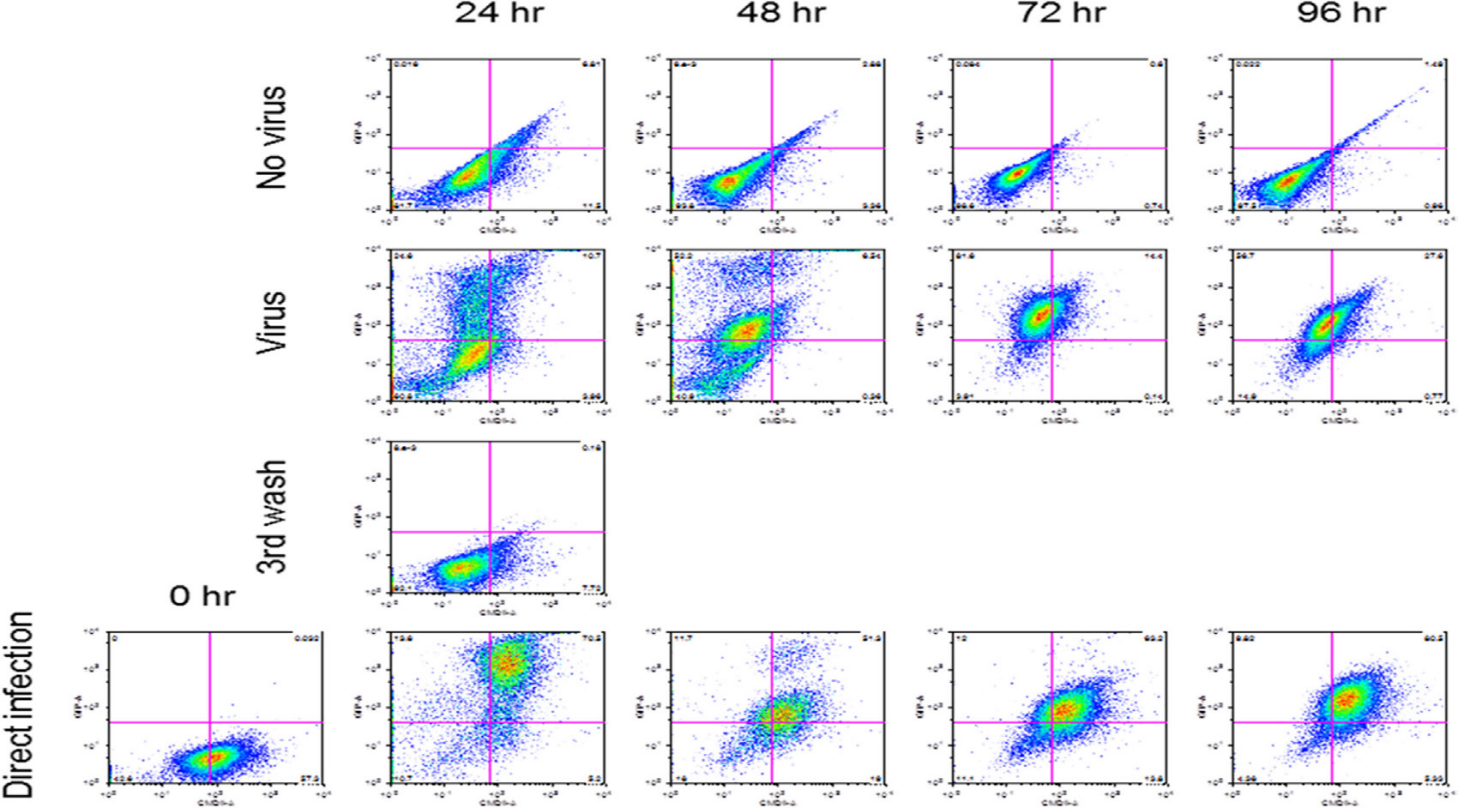
**SSRBCs transfer vesicular stomatitis virus to melanoma cells**. Human sickle RBC were incubated with or without VSV-GFP (MOI = 1) for 2 h at 37°C, washed three times, and then cocultured with B16 cells stained with Vybrant CmDiI at a ratio of 1:1. At indicated time points the B16 cells were washed and analyzed by FACS. As a positive control, B16 cells were directly infected with VSV-GFP at an MOI of 1.

Reovirus is known to be oncolytic, but its delivery to tumor cells is interdicted by immune defenses and neutralizing antibodies (49–51). We therefore determined whether SSRBCs with their known tumor homing properties (29) could serve as vehicles for carriage and delivery of reovirus to tumor and protect it from neutralizing antibodies *in vitro* and *in vivo*. We first examined whether SSRBCs could bind reovirus and vesicular stomatitis virus (VSV) without replicating or inducing SSRBC cytotoxicity. Results shown in Figure 5 indicate that SSRBCs incubated with reovirus or VSV for 1 h at 37°C at various MOIs did not replicate or undergo cytotoxicity in a plaque forming assay. We then determined whether the viral-loaded SSRBCs could transfer reovirus and VSV to melanoma cells in the presence or absence of viral neutralizing antibodies. Results shown in Figure 6 demonstrate that when SSRBCs were loaded with reovirus (SSRBC-neo) and cocultured with B16tk melanoma cells, the virus translocated to melanoma cells and induced melanoma cell lysis at 24 h to a degree comparable to that of melanoma cells directly infected with virus in a standard plaque forming assay. The presence or absence of reovirus neutralizing antibody therefore did not abrogate the viral translocation from SSRBCs to melanoma cells and consequent melanoma cell lysis. VSV showed a similar ability to translocate from SSRBCs and infect melanoma cells (Figure 7). Results demonstrate that, even in the presence of neutralizing antibodies, SSRBCs efficiently adsorb and transfer reovirus and VSV to B16 melanoma cells resulting in tumor cell cytolysis.

### SSRBCs Localize in Melanoma and Loaded with Oncolytic Reovirus Induce a Tumoricidal Response

**Figure 8:**
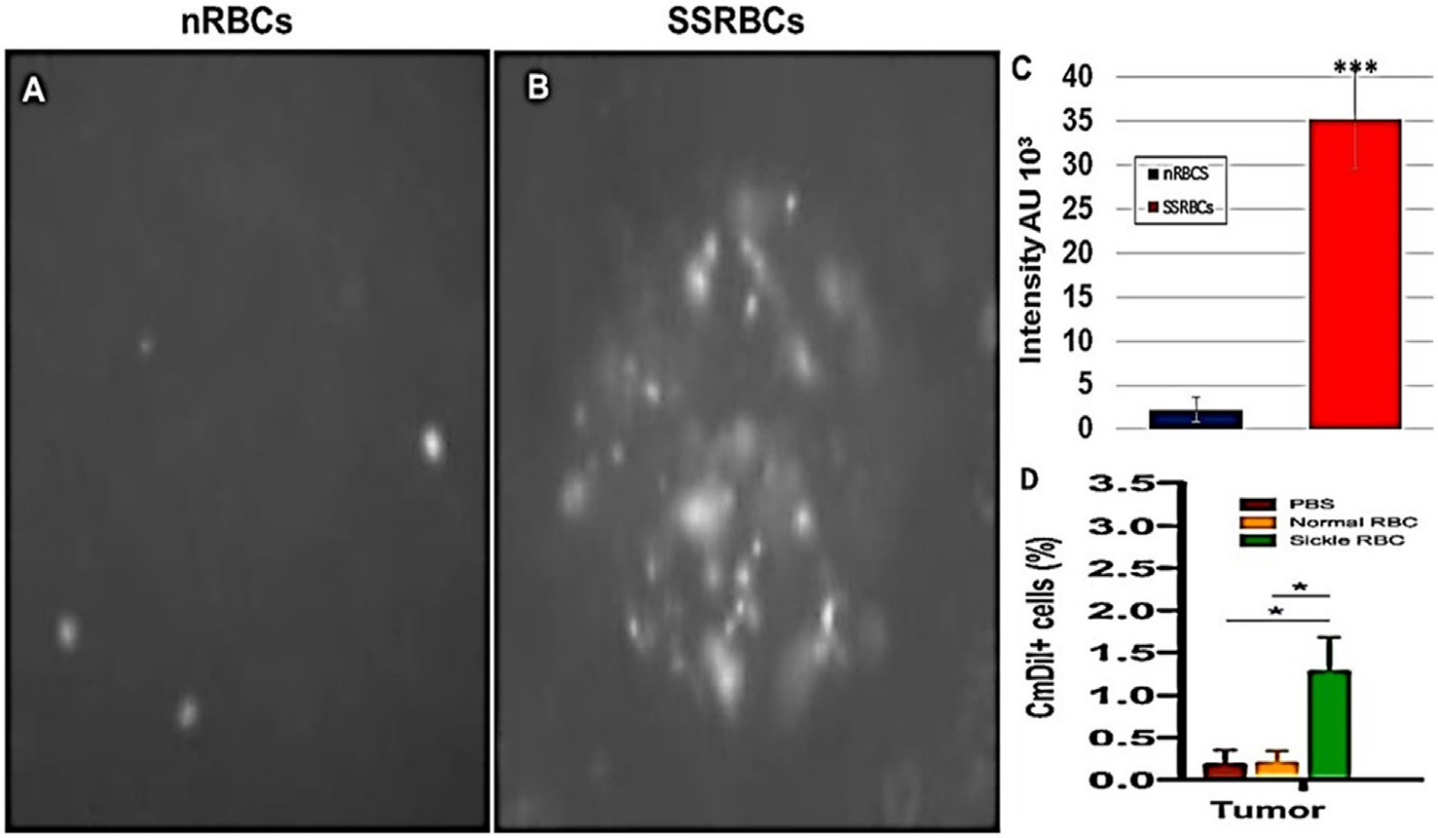
**Sickle cells accumulation in tumor compared to normal RBCs**. Athymic nude mice were injected subcutaneously with 2 × 10^5^ B16tk melanoma cells. When tumors were palpable, nRBCs or SSRBCs stained with Vybrant CmDiI (70% hematocrit) were injected intravenously. Thirty minutes after injection, tumors were harvested and converted to single cell suspensions. Still image of CmDIl uptake in representative tumor suspensions of nRBCs **(A)** and SSRBCs **(B)** from a tumor bearing mouse is shown. Image analysis of **(A,B)** shown in **(C)** indicates a greater number of CmDil staining SSRBCs than nRBCs (*p* < 0.001). FACS analysis of suspensions **(D)** confirmed the increased uptake of CmDil-staining SSRBCs relative to nRBCs in the melanomas (*n* = 3) (*p* < 0.05).

**Figure 9:**
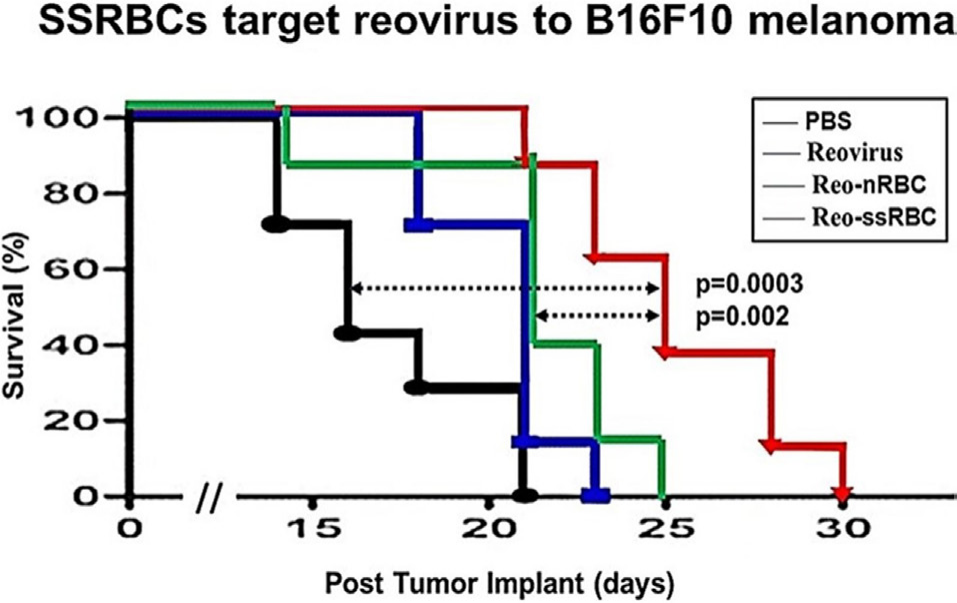
**SSRBCs loaded with reovirus significantly improve the survival of mice bearing established B16 melanoma**. Athymic nude mice were subcutaneously injected in the flank with 2 × 10^5^ B16ova cells. Once tumors were palpable, nRBC or SSRBC were loaded with reovirus for 2 h at 37°C, washed three times and intravenously injected at a hematocrit of 70%. As controls, mice were also intravenously injected with PBS or reovirus (5 × 10^8^ TCID50). Naked reovirus and reo-nRBC prolonged the survival of B16ova-bearing mice compared to the PBS control (*p* = 0.0434 for reovirus alone; *p* = 0.0213 for reo-nRBC). However, reo-SSRBC achieved a highly significant survival enhancement compared to the other three treatment groups (PBS, *p* = 0.0003; naked reovirus, *p* = 0.0020; reo-nRBC, *p* = 0.020).

We compared the ability of intravenously administered SSRBCs and nRBCs to localize in melanomas. Results shown in Figure 8 indicate that 30 min after injection SSRBCs accumulated in melanoma to a degree significantly greater than nRBCs. We then compared the ability of SSRBCs and nRBCs carrying oncolytic reovirus to induce a tumoricidal response in a B16 melanoma model. For this purpose, SSRBCs or nRBCs were loaded with reovirus as described in “Materials and Methods” and injected into mice bearing established B16 melanoma. As controls, mice were also injected with naked reovirus (5 × 10^8^ TCID50) or PBS. Survival of mice receiving SSRBCs-reo was significantly prolonged compared to groups receiving nRBC-reo (*p* = 0.02), naked virus (*p* = 0.002), or PBS (*p* = 0.0003) (Figure 9). Mice showed no significant acute toxicity or weight loss during the study. These data demonstrate that reovirus-loaded SSRBCs can be delivered to melanoma *in vivo* and induce a tumoricidal response exceeding that of the nRBC-reo, PBS control and naked reovirus without significant acute toxicity.

## Discussion

Here, we show that melanoma outgrowth is impaired significantly in transgenic SS knockin mice relative to C57BL/6, B6129SF1/J, and B6129SF2/J background controls and transgenic hemoglobin AA mice. The B16F10 melanoma is syngeneic in C57BL/6 mice but grows comparably in B6129SF1/J and B6129SF2/J mice, which express 129 genes together with B6 genes. This suggests that the 129 genetic component did not interfere significantly with melanoma outgrowth. Likewise, SS and AA mice not only express 129 and B6 genes but also synthesize human hemoglobin allele(s). Since tumor outgrowth occurred in a significantly higher percentage of hemoglobin AA mice relative to SS mice, the presence of the human hemoglobin AA alleles did not appear to interfere significantly with tumor outgrowth or tumor incidence. The restricted tumor outgrowth in SS mice can therefore be explained by the presence of hemoglobin SS alleles in SS knockin rather than by genetic differences between the strains.

As shown herein, impaired melanoma outgrowth in sickle cell knockin mice was accompanied by significant anemia, reticulocytosis, and leukocytosis relative to controls. In sickle cell knockin mice, reticulocytosis is an established marker of underlying chronic hemolysis, while leukocytosis is indicative of a chronic thrombo-inflammatory microvasculopathy induced by recruitment of neutrophils and monocytes to sites of vaso-occlusion (14, 48, 49). In these mice, hemolysis is a consequence of repeated episodes of oxidatively stressful sickling/unsickling, spontaneous SS hemoglobin auto-oxidation, and recurrent episodes of ischemia–reperfusion injury (19, 52, 53). The latter activates vascular pro-oxidant mitochondrial networks (19), while hemolytic by-products arginase and heme reduce bioavailability of antioxidant nitrous oxide (13). SS heme, also activates nuclear factor-kB (Nf-kB) leading to expression of endothelial adhesins VCAM-1, ICAM-1 that bind cognate ligands on SSRBCs leading to formation of cellular microaggregates, blood stasis, vaso-occlusion, and organ injury (54, 55). Figure 4 showing clusters of tumor venules occluded by SSRBCs with surrounding tumor cell necrosis depicts the late phase of tumor microvascular injury. Hemolysis and leukocytosis in SS knockin mice, denoted herein by reticulocytosis and leukocytosis, therefore, play a central role in the biogenesis of the thrombo-inflammatory microvasculopathy. Such an abnormal vascular microenvironment is the likely basis of melanoma growth inhibition in SS knockin mice.

In both SCA and sickle knockin mice, ROS generation results from unrelenting ischemia–reperfusion and nitrous oxide insufficiency. This leads to elaboration of microvascular pro-oxidants NADH, endothelial xanthine oxidase, and peroxynitrites (16, 54). Excessive mitochondrial ROS generated during reperfusion depolarizes mitochondrial membranes (56–59) while microvascular ROS signaling leads to endothelial activation. Intravital microscopy of activated vascular networks in sickle cell mice and solid tumors perfused with SSRBCs shows that microvascular blood flow stagnates leading to vaso-occlusion and autohemolysis with consequent release of excessive ROS (10–12, 29). This self-perpetuating pro-oxidant network is a major determinant of the microvascular injury in SCA and the tumor endothelium (12, 19, 20, 29).

The notion that this highly pro-oxidative microvasculature in SS knockin mice is antithetical to neoangiogensis after implantation of the B16F10 melanoma is supported by recent studies showing that powerful pro-oxidants doxorubicin and zinc protoporphyrin potentiate the tumoricidal effect of SSRBC infusions against the drug resistant, triple negative 4T1 breast carcinoma (29). In addition, SSRBC pro-oxidant surrogates hemin and H_2_O_2_ were shown to induce tumor cell cytotoxicity *in vitro* suggesting that heme, ROS, and heme-nitrosyl complexes generated by interaction of trapped SSRBCs with tumor endothelium are capable of lysing adjacent tumor cells (29). Okuno et al. demonstrated that silencing of the endothelial cell antioxidant mATM gene produced a pro-oxidative microvasculature, which suppressed B16F10 angiogenesis and significantly delayed tumor outgrowth (60). Furthermore, inhibition of GSH and thioredoxin antioxidant pathways or selective activation of the Akt pro-oxidant pathways led to synergistic tumor regression confirming the remarkable potency of excessive ROS when harnessed for tumor eradication (61, 62). In this context, melanoma cells, in particular, have shown an increased sensitivity to exogenous H_2_O_2_ relative to other solid tumor cells (63).

In light of these data, it is likely that melanoma angiogenesis is interrupted in the pro-oxidative vascular microenvironment of sickle knockin mice. During angiogenesis, embryonic tumor blood vessels rely on host endothelial cells to sprout and grow, promoted by VEGF and stabilized by interactions with the extracellular matrix (64, 65). VEGF receptors are activated early in tumor neoangiogenesis and their expression and activation may be impaired in the pro-oxidative vascular milieu of the SS knockin mice (64, 65). Indeed, Subtinib a biologic that competitively blocks VEGF receptors inhibited B16F10 melanoma outgrowth (66). Circulating SSRBCs are likely trapped in the network of sprouting tumor microvessels. Heme and ROS generated from such trapped/hemolyzed SSRBCs may promote free radical generation resulting in tumor endothelial injury, vaso-occlusion, and regression of neoangiogenesis. It is therefore plausible that melanoma neoangiogenesis requiring a coordinated interaction between endothelial cells of SS knockin mice and B16F10 melanoma cells is compromised in the pro-oxidative, thrombo-inflammatory microvascular environment of SS knockin mice.

In related studies, we assessed the ability of SSRBCs to act as a therapeutic to carry and deliver oncolytic reovirus to tumors and induce a tumoricidal effect. Previously, we showed that SSRBCs could be loaded with and release fourfold more drug into hypoxic tumors than free drug or similarly loaded nRBCs (53). Some viral carriers have shown an ability to localize in tumors and induce a tumoricidal effect, while others are hampered by their tendency to sequester in liver, lungs, and reticuloendothelial system (42, 67–69). Although a proportion of virus-loaded SSRBCs are also removed by non-tumor organs, we show here that a larger fraction homes to tumors and extends survival relative to nRBCs and free virus. SSRBCs therefore appear to direct the virus to the tumor where it induces a tumoricidal response.

We further show that reovirus loaded onto SSRBCs can spontaneously translocate to melanoma cells. This transfer may be explained in part by the presence on melanoma cells of high-affinity sialic acid residues along with JAM-A receptors, which promote dissociation of reovirus from its low affinity receptor on SSRBCs (70–72). Notably, adherence of the reovirus to SSRBCs does not kill these cells whereas viral binding to melanoma cells after viral transfer results in melanoma cytolysis. Primary viral adhesion to melanoma cells is followed by a secondary higher affinity interaction between the sigma head of the virus and JAM-A receptors, which promotes virus internalization and melanoma cell apoptosis (73). That neutralizing antibodies did not displace the bound virus from the SSRBCs or the melanoma cells may likewise be ascribed to a higher affinity of the virus for SSRBCs or melanoma cells. Alternatively, the dominant reovirus sigma head epitope recognized by neutralizing antibodies may be sterically screened from recognition by the topology of the virus when bound to SSRBC receptors.

We previously reported that infusion of human SSRBCs along with pro-oxidants into mice bearing established 4T1 breast carcinomas resulted in a tumoricidal response without significant toxicity or histopathologic tissue injury (29). In this context sections of all organs including brains of the treated mice were devoid of inflammation, thrombosis, or necrosis. The lack of toxicity of SSRBC infusions in non-SS tumor bearing mice may be explained by the absence of a procoagulant and inflammatory vascular phenotype in non-tumor organs and repeated cycles of ischemia–reperfusion that predispose SS knockin mice and SCA patients to vaso-occlusion.

Since SSRBCs largely target hypoxic vasculature with upregulated adhesion receptors, cancer patients with vascular diseases bearing an underlying chronic oxidative stress signature such as hypertension and atherosclerotic cardio- and cerebrovascular disease (74) might be at risk higher risk of SSRBC treatment. However, the risk in these settings may be no greater than that of many widely used cancer cytotoxics (e.g., doxorubicin), antiangiogenic agents (e.g., bevacizumab), and biologics (e.g., IL-2), which are known to exacerbate the same vascular conditions. The risk assessment protocol for prospective patients receiving SSRBC treatment would be similar to that used for patients at risk of chemotherapy-associated venous thromboembolism. Particular attention would be directed to parameters predictive of an inflammatory and procoagulant phenotype such as chronic leukocytosis with increased monocytes and PMNs along with high levels of acute phase proteins and cytokines to include TNFα, IL1β, IL6, IL8, IFNγ, CRP, CCL3, CCL2, lactoferrin/elastase, thrombin-anti-thrombin complexes (TAT), and D-dimer (75–80). With appropriate precautions, SSRBC infusions may therefore be useful against several human tumor types without undue toxicity.

In conclusion, these data provide two hitherto unrecognized properties of SSRBCs. First, SS knockin mice restrict melanoma outgrowth relative to background and beta globin controls suggesting that their pro-oxidative microenvironment may constitute a natural host defense system against this tumor. The dissection of this network is likely to lead to pharmacologic mimicry and therapeutic integration of the relevant pro-oxidant systems for prevention and treatment of melanoma. Second, we show that the SSRBC component of this system can function as a therapeutic in its ability to carry and transfer oncolytic reovirus to melanoma cells and induce a tumoricidal response against established melanoma *in vivo*. SSRBCs and the natural microvasculopathic environment of SCA therefore provide novel insight into a potent natural anticancer network and offer promising new tools that can be harnessed for prevention and treatment malignant melanoma.

## Materials and Methods

### Mice

All animal procedures were approved by the UAB and Mayo Foundation Institutional Animal Care and Use Committees or the Animal Use Committees in compliance with the Guide for the Care and Use of Laboratory Animals. Male and female mice 8–12 weeks of age weighing 19–26 g were used. Hemoglobin SS knockin mice (B6; 129-Hba^tm1(HBA/Tow)^ Hbb^tm2(HBG1,HBB)/Tow^/Hbb^tm3(HBG1,HBB)Tow^/J) and homozygous hemoglobin AA knockin mice were obtained from a breeding colony maintained at UAB animal research facility. QTL evaluation of SS and AA knockin mice indicated that they are predominantly B6 and exhibit up to 30% of 129 genes. C57BL/6, B6129SF1/J, and B6129SF2/J mice were purchased from Jackson (Bar Harbor, Me). Female athymic homozygous nude mice (nu^−^/nu^–^), between 8–12 weeks of age weighing 19–26 g, were obtained from Charles River Laboratories (Wilmington, MA, USA) or Harlan Laboratories (Indianapolis, IN, USA). The mice were housed five animals per cage in a 12-h light–dark cycle with water, food *ad libitum*. All infusions were performed using the retro-orbital route or tail vein.

### Tumors, Cells, and Viruses

The B16/F10 melanoma (CRL-6475) was obtained from ATCC, Manassas, VA, USA. B16ova murine melanoma cell line was grown in Dubelcco’s modified Eagle medium supplemented with 10% FBS and 5 mg/mL of G418. B16ova cells were derived from the parental cells by transduction of a cDNA encoding chicken ovalbumin gene. B16tk cells were derived from B16 cells by transducing them with a cDNA encoding the herpes simplex virus thymidine kinase (tk) gene (81) and were grown in DMEM supplemented with 10% FBS and 1.25 μg/mL puromycin. All cell lines were monitored routinely and found to be free of mycoplasma infection. Reovirus Dearing Type 3 was provided by Oncolytics Biotech Inc. (Calgary, AB, Canada). Vesicular stomatitis virus (VSV) was generated as previously described (82). Virus titers were measured by a standard plaque assay on L929 cells for reovirus and BHK-21 cells for VSV.

### Tumor Outgrowth and Established Tumor Studies

Mice were injected in the right flank with 1 × 10^5^ B16F10 melanoma cells in volume of 50 μL containing 25 μL of Matrigel (Fisher Scientific). For the studies in mice bearing B16F10 melanoma, tumor volumes were measured with standard calipers and volumes were calculated as length × width^2^/2 where length is the long axis and width the short axis. Mice were monitored for toxicity. During the 32 day observation period, a tumor was considered to show progressive growth only if its volume exceeded 250 mm^3^. The end point was a tumor volume of 750–1500 mm^3^.

With the approval by the Institutional Review Board of Mayo Foundation normal and sickle red blood cells were obtained from patients with homozygous SCA or normal healthy volunteers. Informed consent was obtained from each donor. For B16ova melanoma studies, subcutaneous (s.c.) tumors were established by injecting 2 × 10^5^ or 5 × 10^5^ B16ova melanoma cells into the flank of C57Bl/6 mice. SSRBC or nRBC infusion studies were started when s.c. tumors reached approximately 200 mm^3^. SSRBCs or nRBCs were loaded with reovirus for 2 h at 37°C, washed three times, and injected intravenously at a hematocrit of 70% in 100 μL. Control mice were injected with PBS or free reovirus (5 × 10^8^ TCID50). Prior to injection, cell smears were stained with DiffQuick, examined under a light microscope, and photographed to ensure sickle cell or normal erythrocyte morphology. Tumors were measured twice a week with standard calipers, and mice were monitored for toxicity. Mice were euthanized if toxicity was evident or tumor burden exceeded 1500 mm^3^.

### Viral Loading of SSRBCs and nRBCs

For the survival studies, human (nude mice) or murine (C57Bl/6 mice) nRBC or SSRBC were loaded with reovirus for 2 h at 37°C, washed three times, and injected intravenously at a hematocrit of 70%. As controls, mice were also injected intravenously with PBS or reovirus (5 × 10^8^ TCID50). Prior to injection, cell smears were stained with Diff-Quick and examined under a light microscope to ensure that sickle cell or normal RBC morphology was maintained.

### Viral Transfer Studies from SSRBCs to Melanoma Cells *In Vitro*

Sickle cells (SSRBC) were incubated with reovirus (MOI = 1) for 2 h at 37°C, washed three times, and then cocultured with B16tk at a ratio of 1:1. In addition, during coculture of reovirus with SSRBC, neutralizing antibody was added to selected wells. In order to demonstrate efficient removal of any unbound virus from the loaded SSRBC, an aliquot of the third wash was directly added to the B16tk cells. Additionally, B16tk cells were directly infected with reovirus at an MOI of 1 for comparison of B16tk cells incubated with and without reovirus neutralizing antibody. At specified time points, aliquots of the supernatant were collected and assayed on L929 cells in a standard plaque forming unit assay.

### MTT Assay

MTT [3-(4, 5-dimethylthiazol-2-yl)-2,5-diphenyl tetrazolium bromide] assays were performed as follows: Target cells were plated in quadruplicate on 96-well plates and were infected with reovirus at various MOIs (ranging from 0.1 to 100). Cell viability was assessed at the indicated time points according to manufacturer’s protocol (Promega, Madison, WI, USA). Optical density was read at 570 nm using an enzyme-linked immunosorbent assay plate reader corrected for background.

### Tumor Uptake Studies

Athymic nude mice were injected subcutaneously with 2 × 10^5^ B16tk melanoma cells. When tumors were palpable, nRBCs or SSRBCs stained with Vybrant CmDiI (70% hematocrit) were injected intravenously. Thirty minutes after injection tumors were harvested and tumor cells were dissociated into single cell suspensions and photographed. Fluorescence intensity of still images was quantified using Adobe Photoshop CS2 software (Adobe Systems Inc., San Jose, CA, USA). Ten determinations of pixel intensity from four quadrants of the image were obtained and averaged to yield mean fluorescence intensity. The difference in uptake of the dissociated cells was confirmed by FACS analysis.

### Hematology

Reticulocyte, leukocyte, and hemoglobin determinations were carried out on tail vein or retro-orbital samples of mouse blood using a Heska HemaTrue analyzer (Loveland, CO, USA).

### Histology

Sections of tumors were stained with hematoxylin and eosin.

### Statistical Analyses

For tumor outgrowth studies, tumor growth curves were analyzed and compared by the method of Kruskal Wallis. Survival curves were plotted according to the Kaplan–Meier method, and statistical significance between the different treatment groups was determined using the log-rank (Mantel–Cox) and Gehan–Breslow–Wilcoxon tests. For comparison of individual data points, two-sided Student’s *t*-test was applied to determine statistical significance.

## Author Contributions

Study concept and design: DT and RV. Acquisition of data: CS, CW, L-CW, JT, and PK. Analysis of data: DT, RV, TT, CS, and CW. Drafting of manuscript: DT. Study supervision: DT and RV.

## Conflict of Interest Statement

The authors declare that the research was conducted in the absence of any commercial or financial relationships that could be construed as a potential conflict of interest.
